# Successful treatment of high-risk myelodysplastic syndrome with decitabine-based chemotherapy followed by haploidentical lymphocyte infusion: A case report and literature review

**DOI:** 10.1097/MD.0000000000010434

**Published:** 2018-04-20

**Authors:** Yuanyuan Ma, Jianliang Shen, Li-Xin Wang

**Affiliations:** Department of Hematology, Navy General Hospital of PLA, Beijing, China.

**Keywords:** decitabine, haploidentical lymphocyte infusion, myelodysplastic syndromes

## Abstract

**Rationale:**

The current therapy for elderly patients with high-risk myelodysplastic syndromes (MDSs) remains unsatisfactory. Decitabine, which has been approved to treat MDS, cannot eliminate malignant clones of MDS.

**Patient concerns:**

A 68-year-old woman presented with multiple divergent bleeding points in the subcutaneous tissue of the limb. Two years earlier, she had been diagnosed with invasive ductal carcinoma of the left breast and had undergone left modified radical mastectomy and local radiation therapy.

**Diagnoses:**

The patient was diagnosed with MDS refractory anemia with excess of blast II and was classified as very high risk according to the revised international prognostic scoring system.

**Interventions:**

The chemotherapy regimen consisted of decitabine (20 mg/m^2^ intravenously on days 1–5), cytarabine (10 mg/m^2^ every 12 hours subcutaneously on days 1–5), aclarubicin hydrochloride (20 mg intravenously on days 1, 3, and 5), and recombinant human granulocyte colony-stimulating factor (250 μg/d subcutaneously from day 0 to day 5). Peripheral mononuclear cells from her son were infused at 36 hours after the end of each chemotherapy cycle. The patient received a total of 4 cycles of the therapy.

**Outcomes:**

The patient achieved complete remission after the first cycle of the treatment. There was no clinical evidence of MDS relapse as of 4 years after the completion of the treatment.

**Lessons:**

The results suggested that decitabine-based chemotherapy and haploidentical lymphocyte infusion may be act synergistically. Combination therapy is a suitable, safe, and effective treatment regimen for elderly patients with high-risk MDS.

## 1 Introduction

Myelodysplastic syndrome (MDS) is a heterogeneous group of clonal myeloid disorders that originate from hematopoietic stem cells. MDS is characterized by dysplastic hematopoiesis of myeloid cell lines, exhibiting single or multiple peripheral blood cytopenias. MDS mainly affects the elderly, who generally have various cardiopulmonary morbidities and poor immune system performance, and they accordingly cannot tolerate intensive chemotherapy. High-risk MDS is generally progressive in nature and can be easily transferred into acute myeloid leukemia (AML). At present, allogeneic hematopoietic stem cell transplantation (alloHSCT) is considered the only curative therapy for the patients with high-risk MDS, but for the elderly it is usually unsuitable. The current therapy for elderly patients with high-risk MDS still remains unsatisfactory. Epigenetic deregulation plays an important role in the pathogenesis of MDS.^[1]^ Several clinical trials have indicated that low-dose decitabine improves outcomes in elderly patients with MDS, including overall survival (OS) and time to AML transformation.^[2,3]^ However, decitabine monotherapy has limited anti-leukemia activity and a relatively low rate of complete remission (CR).^[4]^ These patients require a novel decitabine-based combined regimen. Here, we present a 68-year-old patient with high-risk MDS who received combination therapy consisting of low-dose decitabine, cytarabine, aclarubicin, granulocyte colony-stimulating factor (G-CSF), and human leukocyte antigen (HLA) haploidentical lymphocyte infusion (HLI).

## 2 Case presentation

**Figure 1:**
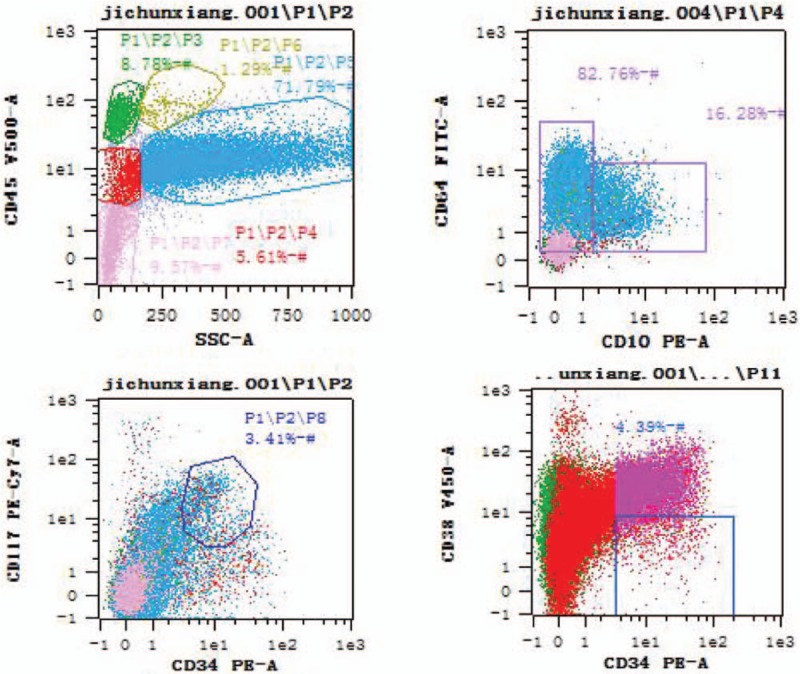
Flow cytometric immunophenotyping graphs of bone marrow before treatment. Mature lymphocytes (P3) comprise 8.78%, myeloid cells comprise 71.79%. CD10^+^ mature granulocytes comprise a relatively low 16.28%. The percentage of CD34^+^ and CD117^+^ blast cells was 3.03%. The percentage of CD10^+^ and CD19^+^ lymphocytes among CD19^+^ B cells was 28.35%.

A 68-year-old woman who presented with no incentive multiple divergent bleeding points in the subcutaneous tissue of the limb for 5 days was admitted to our hospital in May 2013. She was diagnosed with left breast invasive ductal carcinoma in March 2011 and received left modified radical mastectomy in the Xianghe Hospital of Traditional Chinese Medicine in June 2011. Adjuvant chemotherapy with 6 cycles was administered after surgery. Local radiation therapy was performed for a total dose of 5000 cGy from March 5 to April 9, 2012. She reported no history of hypertension, diabetes mellitus, or infectious disease such as diarrhea and hepatitis. She also had no drug allergies or special family history. The routine blood test revealed a white blood cell count of 2.2 × 10^9^/L, a hemoglobin level of 88 g/L, a platelet count of 20 × 10^9^/L and an absolute neutrophil count of 1.3 × 10^9^/L. A bone marrow morphological assay demonstrated 19% blast cells with Auer rods. Chromosome analysis showed a karyotype of 45X, -X.^[5]^ The genetic test indicated a PRAME/ABL ratio of 22%. Immunophenotyping revealed 71.79% myeloid cells, reduced side scatter, decreased percentage of CD10^+^ mature granulocytes (16.2%), elevated proportion of CD15-CD11b-HLA-CR^+^ cells, abnormal CD16/CD13 pattern and increased proportion of CD34^+^ and CD117^+^ immature cells (3.03%; Fig. 1). The patient was diagnosed with MDS refractory anemia with excess of blast II and was classified as very high risk according to the revised international prognostic scoring system. We treated the patient with decitabine-based chemotherapy followed by HLI. The protocol was approved by the Human Ethics Committees of the Chinese Navy General Hospital. The chemotherapy regimen consisted of decitabine (20 mg/m^2^ intravenously on days 1–5), cytarabine (10 mg/m^2^ every 12 hours subcutaneously on days 1–5), aclarubicin hydrochloride (20 mg intravenously on days 1, 3, and 5), and recombinant human G-CSF (250 μg/d subcutaneously from day 0 to day 5). Peripheral blood obtained from her son was processed for separation of mononuclear cells at 36 hours after the end of each chemotherapy cycle using the Baxter CS-3000 Plus blood cell separator. Haploidentical lymphocyte infusion (HLI; an average infused mononuclear cell of 1.66 × 10^8^/kg) was administered. The patient received 1 cycle of treatment per month for a total of 4 cycles. Supportive therapy including erythrocyte suspension and apheresis platelet transfusion was provided. Changes in the expression of epigenetic-related molecules CD80, CD86, CD54, HLA-DR, and HLA-A were detected before and 1 day after the completion of the chemotherapy by flow cytometry. Adverse reactions were evaluated according to WHO anticancer drug and acute toxicity grading standards.^[6]^ After the first cycle of therapy, the patient presented with IV degree of bone marrow suppression, agranulocytosis, and exacerbated pulmonary infection. Then she was treated with intensive supportive care and anti-infective therapy with imipenem, teicoplanin, and itraconazole. As a result, her body temperature gradually returned to normal, and the symptoms of pulmonary infection disappeared. Before the second cycle of treatment, a bone marrow morphology test showed 1.5% myeloblasts suggesting that the patient had achieved CR. The expression of costimulatory molecules CD80 (20.6% vs 40.3%) and CD86 (6.3% vs 12.4%) was significantly greater in malignant cells, and no significant changes in the expression of HLA-ABC and HLA-DR were found before or after treatment. Then the second cycle of therapy was performed and went well. After treatment, the patient experienced moderate constipation but otherwise the patient showed improvement of symptoms after medication treatment. The patient continued to complete the 2 subsequent cycles of treatment. The patient received no other treatment after the final cycle of the combination therapy. There was no clinical evidence of MDS relapse as of 4 years after the completion of this regimen. The patient signed written informed consent and consent for publication.

## 3 Discussion

Elderly patients with high-risk MDS tend to have poor prognosis, and there remains no consensus regarding the optimal therapy. We treated a patient with very high-risk MDS with decitabine-based chemotherapy followed by HLI. She achieved CR after 1 cycle of treatment and received 3 more cycles for consolidation. There was no sign of relapse as of the consequent 4-year follow-up. During the first cycle of treatment, CD80 and CD86 on the surface of blast cells were increased by decitabine treatment, which was consistent with our previous study.^[7]^ CD80 and CD86 were the strongest costimulatory molecules. Higher expression of CD80 and CD86 on blast cells provides stronger signal to stimulate cytotoxic T cells.^[8]^ HLI was given 36 hours after the completion of decitabine in our combination therapy. The results suggested that decitabine and the haploidentical lymphocytes may act synergistically in the eradication of malignant cells.

Decitabine, a demethylating agent, was approved for treatment of MDS in 2006^[9]^ and it has also been used to treat myeloid malignancy in many clinical trials.^[5,10]^ However, little attention has been paid to its effect on modulating the immune system. Recently, accumulating evidence has demonstrated that decitabine can enhance the immunogenicity of leukemic cells by inducing the expression of cancer testis antigen,^[11]^ major histocompatibility complex molecules, and costimulatory molecules.^[7,12]^ Two articles published in the same issue of *Cell* showed that demethylating agent rendered that cancer cells more immunogenic.^[13,14]^ The combination of epigenetic drug and immunotherapy achieved radical improvement in patient outcome. Several years ago, we designed a combination treatment including decitabine-based chemotherapy and HLI. Four years ago, we performed a clinical trial to treat elderly patients with AML. The clinical trial included 29 elderly patients (median age: 64 years, range 57–77 years) with AML. The overall CR rate was 72.4%. The 2-year OS and disease-free survival were 59.6% and 36.9%, respectively.^[15]^ The results were much better than those of conventional chemotherapy, which suggested that the demethylating agent might act synergistically with the following HLI. Recently, more and more studies have demonstrated that epigenetic therapy might render the malignant cells more vulnerable and sensitive to subsequent immunotherapy.^[16–18]^ The rationale of synergy of demethylating agent and immunotherapy provided a promising option for the treatment of malignancy.

Because the current work is a single case report, large-scale clinical trials are warranted to confirm this conclusion. Combination therapy was here shown to be tolerable and suitable for the treatment of elderly patients. In conclusion, a combination of decitabine-based chemotherapy and HLI may be a suitable, effective, and safe treatment regimen for elderly patients with high-risk MDS. This merits further study.

## Author contributions

**Data curation:** Yuanyuan Ma.

**Investigation:** Jianliang Shen.

**Supervision:** Jianliang Shen.

**Writing – original draft:** Li-Xin Wang.

**Writing – review & editing:** Li-Xin Wang.
